# A microRNA-based signature predicts local-regional failure and overall survival after pancreatic cancer resection

**DOI:** 10.18632/oncotarget.27496

**Published:** 2020-03-10

**Authors:** Adam R. Wolfe, Patrick Wald, Amy Webb, Nikhil Sebastian, Steve Walston, Ryan Robb, Wei Chen, Marall Vedaie, Mary Dillhoff, Wendy L. Frankel, Wooil Kwon, Jin-Young Jang, Terence M. Williams

**Affiliations:** ^1^ The Ohio State University Medical Center, Arthur G. James Comprehensive Cancer Center and Richard J. Solove Research Institute, Columbus, OH, USA; ^2^ Department of Surgery, Cancer Research Institute, Seoul National University College of Medicine, Seoul, Korea; ^*^ Co-first authors

**Keywords:** pancreatic cancer, miRNA, non-coding RNA, prognostic biomarker, local-regional recurrence

## Abstract

Resectable pancreatic adenocarcinoma (PC) is generally managed with surgery followed by chemotherapy, but the role of postoperative chemoradiation (pCRT) is controversial. We sought to identify a microRNA (miRNA) expression profile associated with higher risk for local-regional recurrence (LRR), which might help identify patients that may benefit from pCRT. Total RNA was isolated from viable tumor from 88 patients who underwent PC resection with or without chemotherapy, but did not receive radiation. Digital miRNA expression profiling was performed and risk scores were calculated based on the expression levels of the four most significantly correlated miRNAs, and dichotomized about the median to detect correlations between risk group, LRR and overall survival (OS). Two cohorts from The Cancer Genome Atlas (TCGA) and Seoul National University (SNU) were used for validation. Patients with high-risk scores had significantly worse LRR (*p* **=** 0.001) and worse OS (*p* **=** 0.034). Two-year OS rates for the high- and low-risk groups were 27.7% and 52.2%, respectively. On multivariable analysis, the risk score remained significantly associated with LRR (*p* **=** 0.018). When validated on TCGA data, a high-risk score was associated with worse OS on univariate (*p* **=** 0.03) and multivariable analysis (*p* **=** 0.017). When validated on the SNU cohort, a high-risk score was likewise associated with worse OS (*p* **=** 0.042). We have developed a 4-miRNA molecular signature that is associated with risk of LRR and OS after PC resection and validated on two separate cohorts. This signature has the potential to select patients most likely to benefit from pCRT, and should be tested further.

## INTRODUCTION

In the United States, there are an estimated 56,770 new cases of pancreatic carcinoma (PC) and 45,750 estimated deaths [1]. Approximately 15–20% of PC patients present early enough to be considered candidates for gross surgical resection [2]. For surgically resectable patients, common standard of practice is surgery followed by adjuvant chemotherapy [3, 4]. Unfortunately, even after radical surgery, prognosis remains poor, although more modern adjuvant chemotherapy trials have shown improvement of median overall survival (OS) to 46-54.4 months [4, 5]. The role of post-operative chemoradiation, typically delivered with 5-fluorouracil chemotherapy over 5–6 weeks, is controversial. Local-regional recurrence (LRR) rates range from 23%-63% after surgery for patients who don’t receive postoperative chemoradiation [6, 7]. However, prior randomized trials attempting to improve progression-free (PFS) and overall survival (OS) with post-operative radiation have yielded conflicting results [8–10]. Interestingly, a National Cancer Database (NCDB) study and retrospective studies from Mayo Clinic and Johns Hopkins have reported superior local-regional control (LRC) and OS in patients receiving adjuvant chemoradiation (CRT) versus observation or chemotherapy (CT) alone [11–13]. Alternatively, a more recent NCDB propensity-matched study of margin negative (R0) and node-positive patients showed no OS improvement with the use of CRT vs CT alone [14]. The latest ASCO guidelines (2016) as well as NCCN recommend the consideration of adjuvant radiotherapy for patients with microscopic residual disease and/or node positive disease [15, 16]. The ongoing phase III RTOG 0848, a randomized trial between adjuvant CRT versus chemotherapy alone, should provide more clarity on whether adjuvant chemoradiation can improve outcomes. Given the potential toxicity of concurrent chemotherapy and radiation, it is important to develop and validate risk classifiers that could predict which patients would benefit most from post-operative chemoradiation.

Past efforts have focused on using clinical and pathologic features (margin status, nodal involvement, post-operative CA 19-9, etc.) to predict patterns of failure and prognosis after surgery, in hopes that improved patient selection for adjuvant therapy may improve outcomes. Recently, molecular profiling of pancreatic cancer is an emerging field with potential to provide valuable tumor-specific information [17, 18]. MicroRNAs (miRNA) are a class of small, non-coding RNA molecules and biomarkers which exhibit oncogenic and tumor suppressor functions in a wide variety of human cancers, including PC [19]. These small, non-coding RNA molecules function via messenger RNA (mRNA) silencing and post-transcriptional gene expression regulation [20]. As we learn more about aberrant miRNA expression and downstream signaling effects, miRNA profiling may have potential to detect malignancy, predict patterns of spread, and provide valuable prognostic information [21–27].

Given the controversial role of post-operative chemoradiation in pancreatic cancer, we sought to develop a molecular test that could predict patients who have increased risk of local-regional recurrence and/or worse overall survival. To that end, we carefully characterized patterns of recurrence in patients treated with surgery and post-operative chemotherapy alone at our institution using patterns of failure radiologic analysis. We then performed molecular profiling of the miRNA-ome using total RNA extracted from tumor cores in formalin-fixed paraffin-embedded tissue previously identified to have viable tumor cores. Our aim is to identify a miRNA expression profile that correlates with LRR and OS after surgical resection which might be used to better select patients who could benefit most from adjuvant chemoradiation in the future. Herein, we describe the generation of a multi-miRNA molecular signature.

## RESULTS

### Patient characteristics

After statistical filtration and normalization of miRNA profiling results, 93 patients were included in our overall survival analysis. Table 1 shows the clinical and pathologic characteristics of the initial cohort. The majority of patients (89/93, 96%) received adjuvant chemotherapy. Median age is 63.5 years (range, 39–88). There are 56 males and 37 females included. The majority of patients had pathologic T3, pathologic N1, grade 2 disease with negative surgical margins and post-operative CA 19-9 levels less than 90.

**Table 1:** Clinical and pathologic characteristics of the OSU cohort

	*n* (%)
**Age**, years, median (range)	63.5 (39–88)
**Gender**	
Male	56 (60)
Female	37 (40)
**pT stage**	
1	2 (2)
2	5 (5)
3	85 (92)
4	1 (1)
**pN stage**	
0	17 (18)
1	76 (82)
**Histologic grade**	
1	4 (4)
2	63 (68)
3	26 (28)
**Surgical margin**	
Positive	21 (23)
Negative	72 (77)
**Post-op CA 19-9**	
< 90	52 (56)
90–180	28 (30)
>180	13 (14)
**Post-operative chemotherapy**	
N/A	4 (4)
Gemcitabine	76 (82)
Gemcitabine/Abraxane	13 (14)
**Anatomic location of tumor**	
Head/Neck	61 (66)
Body/Tail	32 (34)

### Clinical outcomes

Median follow up for surviving patients was 24.6 months. Median overall survival was 19.8 months. At the time of data collection, 75 patients (75/93, 80.7%) were deceased and 18 patients (18/93, 19.3%) were alive. Eighty-eight of these patients had adequate follow up imaging for assessment of date and location of disease recurrence. In total, 47 patients (47/88, 53.4%) developed local-regional recurrence. Of these local-regional recurrences, 37 patients (37/88, 42.0%) developed local recurrence in the surgical bed. Of these 47 local recurrences, 3 patients (6.3%) had a biopsy confirmed recurrence, with the remaining recurrences classified by clinical exam and CT imaging. Twenty-four patients (24/88, 27.3%) developed local-regional recurrence as their first site of recurrence, and of these, 20 (20/88, 22.7%) developed local-regional-only disease, with no evidence of distant metastases. For the 24 patients with local-regional recurrence as the first site of recurrence, 6 (25%) received salvage RT with four patients receiving concurrent 5-FU based chemotherapy and RT (dose ranged from 20–30 Gy) and two patients receiving stereotactic body radiation therapy (SBRT). The remaining patients with recurrences received chemotherapy alone.

Fifty-eight patients (58/88, 65.9%) developed distant metastases, 40 of which (40/88, 45.4%) developed distant metastases within one year after their date of surgery. Fifty patients (50/88, 56.8%) developed distant metastases as their first site of recurrence or simultaneous with a local-regional recurrence. Thirty-one patients (31/88, 35.2%) developed distant recurrence only, with no evidence of local-regional disease. The most common sites of distant metastases were liver (44/88, 50%), lung (16/88, 18.2%), peritoneum (9/88, 10.3%), and bone (6/88, 6.8%). Patterns of survival and disease recurrence are summarized in Table 2.

**Table 2:** Patterns of failure analysis of the OSU cohort

**Current status**	
Alive without disease	8 (9.1)
Alive with disease	8 (9.1)
Deceased without disease	2 (2.2)
Deceased with disease	70 (79.5)
**Sites of recurrence**	
None	12 (13.6)
Local	37 (42.0)
Local-regional	47 (53.4)
Distant	58 (65.9)
**First site of recurrence**	
Local-regional	24 (27.3)
Distant	36 (40.9)
Both	14 (15.9)
**Local-regional or distant predominant**	
Local-regional only	20 (22.7)
Distant only	31 (35.2)
**Time of distant metastasis**	
N/A	30 (34.1)
< 12 months	40 (45.5)
> 12 months	18 (20.5)

### MicroRNA risk score

**Figure 1:**
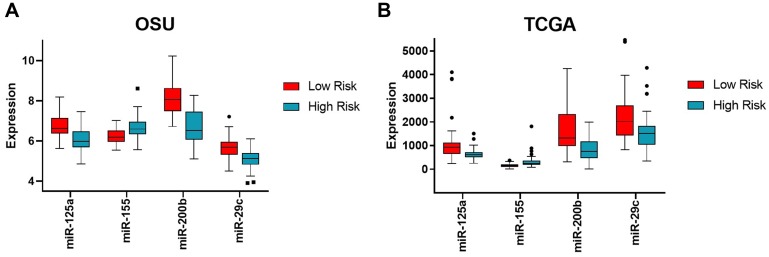
Expression levels of the four miRNAs in high versus low risk groups in the OSU and TCGA cohorts. Expression levels of each miRNA (mir-125a, miR-155, miR-200b, and miR-29c) are shown as boxplots with the median and 95% confidence intervals for each of the binary risk groups (high versus low risk) for both OSU (**A**) and TCGA (**B**) cohorts. Low risk shown in red, high risk shown in cyan. Note the common trend with miR-155 being up-regulated in high risk score while the other three miRNAs being down-regulated in high-risk score between cohorts.

The four most correlative miRNAs (miR-29c, miR-125a, miR-155, and miR-200b) were used to generate patient-specific risk scores based on miRNA expression levels. The range for the miRNA risk score was between -18.3 to -8.7 in the OSU cohort and -33.1 to -14.7 in The Cancer Genome Atlas (TCGA) cohort (Supplementary Figure 1). Dichotimization of these risk scores by the median defined “low risk” versus “high risk” (Figure 1A). As noted in Figure 1A, miR-125a, miR-200b, and miR-29c were up-regulated in low risk tumors, while miR-155 was up-regulated in high risk tumors. We also downloaded miRNA expression data from the TCGA pancreatic cancer study (miR-Seq), and calculated risk scores for each patient in the TCGA study (178 patients). Similarly, the same trends were observed for each of the 4 miRNAs of the risk score (Figure 1B).

### Local-regional control

**Figure 2:**
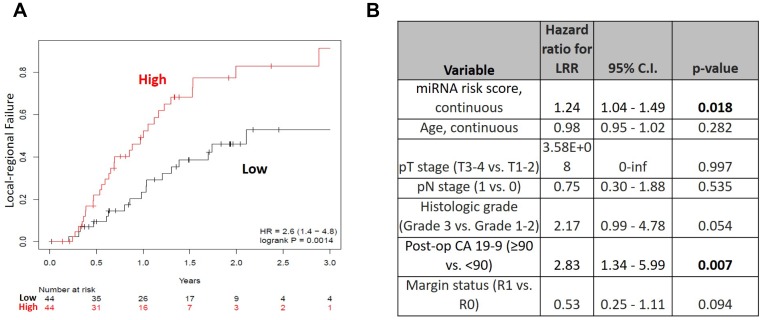
(**A**) Local-regional failure rates over time for high (red) versus low (black) risk groups in the OSU cohort and (**B**) Multivariable analysis for local-regional recurrence (OSU cohort).

The high-risk miRNA signature was significantly associated with LRR (HR 2.6, 95% CI 1.4–4.8, *p =* 0.0014) (Figure 2A). Twenty-nine patients (18/44, 65.9%) in the high risk group developed LR recurrences, compared to eighteen patients (18/44, 40.9%) in the low risk group. The one- and two-year local regional failure rates were 56.8% (25/44) and 36.4% (16/44) vs. 79.5% (35/44) and 63.6% (28/44), for the high and low risk groups respectively. The median times to LRR for the high and low risk groups were 252 days and 367 days, respectively. On multivariable analysis, the high risk signature remained significantly associated with LRR (HR 1.24, 95% CI 1.04–1.49, *p =* 0.018) after accounting for age, pathologic T stage, pathologic N stage, histologic grade, post-op CA 19-9, and surgical margin status (Figure 2B).

### Overall survival

**Figure 3:**
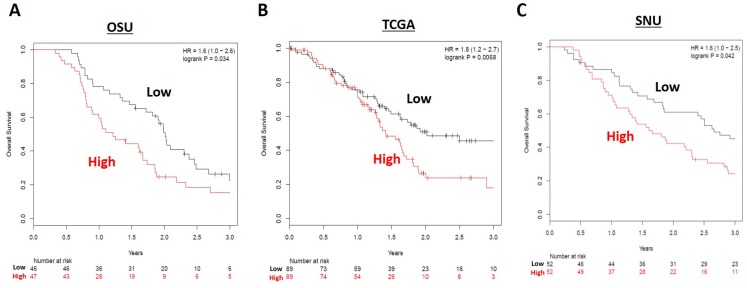
Overall survival (OS) for high (red) versus low (black) risk groups in the (**A**) OSU, (**B**) TCGA, and (**C**) SNU resected cohorts. Patients in the low risk miRNA grouping had longer OS in all three resected patient cohorts.

The high risk miRNA signature was associated with worse overall survival in the OSU cohort (HR 1.6, 95% 1.0–2.6, *p =* 0.034) (Figure 3A). Median overall survival for high and low risk groups were 1.21 years and 1.98 years, respectively. One-year overall survival rates for high and low risk groups were 59.6% (28/47) and 78.3% (36/46), respectively. Two-year overall survival rates for high and low risk groups were 27.7% (13/47) and 52.2% (24/46), respectively. The median times to death for high and low risk groups were 377 days and 664 days, respectively. On multivariable analysis (Table 3), there was no statistically significant association of risk score with overall survival (HR 1.12, 95% CI 0.94-1.33, *p =* 0.197).

**Table 3:** Multivariable analysis for overall survival (OSU and TCGA cohorts)

Variable	Hazard ratio for OS	95% C. I.	*p*-value
**OSU *n*** **= 88**			
miRNA risk score, continuous	1.12	0.94-1.33	0.197
Age, continuous	1.01	0.98-1.05	0.341
pT stage (T3-4 vs. T1-2)	11.17	1.38-90.25	**0.024**
pN stage (1 vs. 0)	0.98	0.43-2.22	0.962
Histologic grade (Grade 3 vs Grade 1-2)	1.45	0.70-3.02	0.317
Post-op CA 19-9 (≥90 vs. <90)	1.71	0.90-3.27	0.102
Margin status (R1 vs. R0)	0.54	0.28-1.04	0.065
**TCGA *n*** **= 178**			
miRNA risk score, continuous	1.14	1.03–1.28	**0.016**
Age, continuous	1.03	1.01–1.05	**0.018**
pT stage (T3-4 vs. T1-2)	1.33	0.65–2.69	0.429
pN stage (1 vs. 0)	1.51	0.83–2.74	0.172
Histologic grade (Grade 3 vs Grade 1-2)	1.13	0.97–3.01	0.595
Margin status (R1 vs. R0)	1.89	1.19–3.0	**0.007**

In the validation set from The Cancer Genome Atlas (*n* = 178), a high risk score was again associated with worse overall survival (HR 1.80, 95% CI 1.2–2.7, *p =* 0.0068) (Figure 3B). On multivariable analysis, a high risk score was significantly associated with overall survival (HR 1.14, 95% CI 1.03-1.28, *p* = 0.016) (Table 3). When TCGA patients who received post-operative radiation or chemoradiation were excluded (to make the cohort more similar to the OSU dataset), there was still an association between high risk score and worse overall survival in the remaining 102 patients (HR 1.8, 95% CI 1.1–3.0, *p =* 0.03) (Supplementary Figure 2). For these group of patients, the result of multivariable analysis for overall survival remained relatively unchanged (HR 1.15, 95% 1.03–1.30, *p =* 0.017) (Supplementary Table 1).

We next obtained survival outcomes for a second validation cohort consisting of 104 pancreatic cancer patients from Seoul National University (SNU) who were treated with surgical resection from 2008–2013 and had previous miRNA profiling performed [28]. In this cohort, 83 patients (83/104, 79.8%) received adjuvant chemotherapy, 60 (57.7%) of which also received concurrent radiation. When our miRNA risk score was tested against the SNU validation cohort, a high risk score was likewise associated with worse overall survival (HR 1.6, 95% CI 1.0–2.5, *p =* 0.042) (Figure 3C).

## DISCUSSION

Despite aggressive surgical resection, localized pancreatic adenocarcinoma is associated with poor outcomes due to high rates of local-regional and distant recurrences. The established therapy for medically-operable and surgically-resectable pancreatic cancer is surgical resection followed by adjuvant chemotherapy. The benefit of adjuvant chemoradiation (CRT) is more controversial and the subject of a recently closed randomized trial (RTOG 0848), for which we are awaiting results. Despite this, several lines of evidence point to the need for better selection of chemoradiation therapy: (1) local-regional recurrence rates for patients who do not receive adjuvant chemoradiation are high; (2) toxicity of chemoradiation is notable; (3) recent evidence suggests that there is a subset of patients with local-regional recurrence predominant disease; and (4) local-regional disease progression can contribute directly to morbidity and mortality [6, 7, 23, 29]. Taken together, this suggests a need to better select patients for aggressive local-regional therapy versus systemic therapy or supportive care alone.

In our cohort of patients treated with surgery followed by chemotherapy alone, approximately half of all patients developed local-regionally recurrent disease. In addition, approximately one out of four patients developed a local-regional recurrence as their first site of failure. Of those, about 80% developed local-regional disease only without distant progression, suggesting that 20-25% of patients have local-regional predominant disease and longer (or no) distant metastasis-free intervals. Conversely, nearly half of our patients developed distant metastases within 12 months of surgery, representing the biologic subset of patients who would likely not benefit from adjuvant chemoradiation. Clinical predictors of recurrence patterns (margin status, lymph node involvement, CA 19-9) can be used to guide adjuvant therapy recommendations, but are limited in their predictive efficacy. Molecular profiling of resection specimens thus has the potential to provide valuable prognostic and predictive information for identifying predictive and prognostic patient subgroups.

To that end, we have identified a molecular-based risk stratification model based on expression levels of four microRNAs in PC resection specimens. The dichotomized risk score identified patients in our cohort who were more likely to develop local-regional recurrence. Additionally, the risk score was also associated with overall survival in our cohort, as well as two validation cohorts including TCGA (178 patients) and Seoul National University (104 patients). This suggests that miRNA profiling can be used to prospectively identify the subset of patients most likely to benefit from adjuvant chemoradiation. The strengths of this study include showing that the risk score predicts for overall survival across different miRNA expression platforms (e. g nanoString, miR-Seq, and GeneChip), and rigorous patterns of failure analysis in the OSU cohort. The limitations of our study include no prospective validation of our miRNA risk score for predicting LRR (given TCGA and SNU cohorts lack patterns of failure data), and the inability to perform multivariable analysis of the Korean dataset due to lack of clinical data.

With regards to the function of the miRNAs in our risk signature, miR-29c, miR-125a, and miR-200b appear to function as tumor suppressors in pancreatic cancer, findings that are supported both by our own data showing that they are down-regulated in the high-risk signature in OSU and TCGA datasets (Figure 1), and from multiple prior publications (Supplementary Table 2). For example, the miR-200 family has been reported as a tumor suppressor in multiple human malignancies, including pancreatic cancer. Yu *et al.* demonstrated that low miR-200c expression was associated with significantly worse overall survival in pancreatic cancer patients after pancreatectomy [30]. Proposed targets of the miR-200 family include ZEB1, ZEB2 [31] and Ezrin-Radixin-Moesin (ERM) [32]. Hong *et al*. showed that aberrantly low expression of miR-200b has an inverse relationship with ERM, resulting in enhanced cell migration and invasion. Park *et al*. demonstrated that downregulated miR-200 reduced E-cadherin expression, thereby inducing epithelial-to-mesenchymal transition [32]. A meta-analysis by Qi *et al*. on the prognostic utility of miR-29 in various human malignancies reports that aberrantly low or absent levels of miR-29 are significantly associated with worse disease-free survival and overall survival [33]. A study by Zhang *et al*. proposes a mechanism of action for miR-29c in non-small cell lung cancer. Their data suggests that miR-29c directly represses specificity protein1 (Sp1), a key protein involved in TGF-β-mediated epithelial-to-mesenchymal transition (EMT) and cell invasion [34]. The theory that miR-125a functions as a tumor suppressor is supported by data from Tang *et al*., who transfected miR-125a mimics into human hepatocellular carcinoma (HCC) cell lines and demonstrated that colony formation and migration rates were decreased in miR-125a upregulated cells. They also report that miR-125a transfection was associated with down-regulation of phosphoinositide 3-kinase (PI3K)/AKT/mammalian target of rapamycin (mTOR) messenger RNA expression, suggesting that miR-125a exerts tumor suppressive effects by targeting the PI3K/AKT/mTOR pathway [35].

Finally, our data supports that miR-155 exhibits oncogenic properties in pancreatic cancer. Interestingly, multiple prior publications have cited miR-155 as an oncogenic miRNA in pancreatic cancer, based on data showing the higher expression levels are associated with worse overall survival [36–38]. A meta-analysis by authors Frampton *et al*. in 2015 analyzed twenty studies with 1525 patients examining individual miRNAs prognostic for OS. High tumoral miR-155 was found in this meta-analysis to predict for worse OS in 3 combined studies with a combined HR of 2.08 (95% CI: 1.26–3.44, *p =* 0.004) [39]. The other three miRNAs in our signature were not found to associate with OS in this study. This highlights the need for combined miRNA signatures compared with single miRNAs to better group patients into high and low risk categories.

Limitations of this study as include the reliability of the genomic data acquired from FFPE samples and the type of technology used to quantify individual miRNAs. FFPE tissue samples can degrade RNA and cross-link nucleic acids and proteins during the process of formalin. Although our tissue was obtained after pathologic review of the tumor sections to find tumor regions enriched with viable tumor cells with less contaminating stroma, the samples obtained during the coring process were not microdissected, so there is likely some combination of stroma in addition to tumor. Therefore, the signature of the 4 miRNAs likely represent a mixture of tumor and stromal miRNAs. With regards to differential expression of miRNAs within tumor and stroma, studies have found miR-155 overexpression and miR-200b down-regulation are more commonly found in tumor cells compared to stroma [38, 40]. In addition, one particular study showed miR-29 was commonly lost in pancreatic stellate cells from pancreatic cancer [41]. In our cohort, we utilized the nanoString nCounter platform which assays ~800 miRNAs. While this assays the vast majority of known human miRNAs, it does leave the possibility that important miRNAs are not assessed. The benefits of using nCounter are the relative lower cost compared to next generation sequencing, the lower amount of RNA input required (only 100 ng vs 1000 ng), the lack of need for technical replicates, and the high success rate of this technology in FFPE tissue. One study compared three miRNA technologies (next generation sequencing, microarray and nanoString) for profiling miRNAs using clinical FFPE samples and found high reproducibility and significant levels of shared detection between the platforms [42].

In summary, we have developed a four miRNA risk score that provides prognostic information for clinical outcomes after surgical resection for pancreatic cancer. Based on the ability of our four miRNA risk score to predict local-regional control, as well as overall survival in three cohorts, such molecular profiles have the potential to help guide clinical decision-making for pancreatic cancer patients after surgical resection. We further intend to apply our risk stratification miRNA score to patients treated on prospective clinical trials with surgery followed by chemotherapy with and without chemoradiation to validate its ability to predict LRR and OS. In addition, this risk score warrants testing on patients who have received neoadjuvant (preoperative) chemotherapy for locally-advanced or borderline resectable PC in order to determine if this miRNA signature can risk-stratify patients in the neoadjuvant setting who would benefit from escalated local-regional therapy (such as radiation or chemoradiation). Such a risk score in the neoadjuvant setting could help decide the need to employ radiation therapy to improve margin negative resection rates and lymph node clearance rates, thereby likely improving local-regional recurrence. Thus, we feel that further validation studies with larger patient numbers and with rigorous patterns of failure data are warranted.

## MATERIALS AND METHODS

### Clinical specimens and study design

This study was approved by the institutional review board of The Ohio State University. We identified 149 adult patients who underwent radical surgical resection of non-metastatic pancreatic ductal adenocarcinoma at The James Comprehensive Cancer Center at The Ohio State University between 2006 and 2014. Patients were allowed to have received post-operative chemotherapy only and were excluded if they received post-operative radiation therapy. Of these patients, 113 had archived formalin-fixed paraffin-embedded (FFPE) blocks of surgical tumor specimens available for RNA extraction and analysis. None of the patients had received neoadjuvant chemotherapy or radiotherapy. After miRNA expression profiling, statistical normalization and filtration resulted in a total of 93 patients with quality miRNA samples and overall survival follow up data. Of those, 88 patients had adequate follow up imaging available to assess patterns of disease recurrence.

### Clinical data collection

Clinical, pathologic, and demographic data was retrospectively collected from electronic medical records, including age, gender, pre-operative CA 19-9, post-operative CA 19-9, clinical stage, pathologic stage, pathologic features, adjuvant therapy, disease recurrence date/location, and overall survival. Pathologic staging was based on the criteria of the American Joint Committee on Cancer staging manual (seventh edition). Patients were routinely followed up by their surgical oncologist and/or medical oncologist every 3-6 months after surgery with computed tomography (CT) and serum CA 19-9.

Disease recurrence was defined as a newly detected mass or soft tissue density on follow up CT, magnetic resonance imaging (MRI), and/or fluorodeoxyglucose positron emission tomography (FDG-PET) imaging. Local recurrence (LR) was defined as a recurrence within the surgical bed. Local-regional recurrence (LRR) was defined as a recurrence occurring within traditional post-operative radiotherapy volumes, including the surgical bed and regional lymphatics. Distant recurrence (DR) was defined as a recurrence occurring outside of a traditional post-operative radiotherapy field. Disease-free survival (DFS) was defined as the time interval from the date of surgery to the date on which a radiographic recurrence was detected. Overall survival (OS) was defined as the interval between the date of surgery and the date of death or last follow up. Patients were followed from their date of surgery until death or censoring, whichever came first.

### miRNA extraction and quantification

Hematoxylin & eosin stained slides of corresponding FFPE blocks were used to mark areas of viable, non-necrotic tumor for coring. Then, 1.75 mm cores were obtained from the FFPE blocks using disposable 14 gauge needles. Total RNA isolation was performed for 113 pancreatic tumor specimens using the Norgen FFPE RNA Isolation kit as previously described [43]. We performed miRNA expression profiling using the nanoString nCounter Human v3 miRNA Expression Assay. A minimum of 100 ng of total RNA was annealed with multiplexed DNA tags (miR-tag) and target-specific bridges. Mature microRNAs were bound to specific miR-tags using a ligase enzyme and all the tags in excess were removed by an enzyme clean-up step. The tagged microRNAs product was diluted 1 to 5, and 5 μL was combined with 20 μL of the Reported Probes in hybridization buffer and 5 μL of Capture probes. The overnight hybridization (16 to 20 hours) at 65° C allowed the probes to complex in a sequence-specific fashion with the targets. Probe excess was removed using two-step magnetic beads-based purification on an automated fluidic handling system (nCounter Prep Station), and target/probe complexes were immobilized on the cartridge for data collection. The nCounter Digital Analyzer collected the data by taking images of immobilized fluorescent reporters in the sample cartridge with a CCD camera through a microscope objective lens. For each cartridge, a high-density scan encompassing 600 fields of view was performed. Expression levels of approximately 800 unique miRNAs in each tissue specimen were recorded.

### Statistical analysis

Filtering of a given miRNA was performed if more than 90% of the samples had log counts less than the negative background. Negative background was calculated as the mean of the log2 negative background counts plus 1.5 times the standard deviation. A sample was removed if more than 70% of miRNA probes fell below the background cutoff. The final filtered data was normalized by the geometric mean and log2 transformed. After statistical filtering of the nanoString data from ~800 miRNAs, 199 miRNAs remained for analysis in 93 tissue samples with sufficient high quality and adequate expression for analysis. Of these, a total of 30 miRNAs were identified to have been commonly linked to pancreatic cancer (listed in Supplementary Table 3) after a publication search. Using this pool of selected miRNAs, elastic net regression was used to identify predictive groups of miRNAs for LRR. Beta coefficients, generated by Elastic net, were used to calculate a risk score by multiplying each miRNA expression level by its beta coefficient and summing all miRNAs in the panel. The risk score was calculated as follows:

-1.0067*miR-29c-1.3221*miR-125a+1.0506*miR-155-0.7891*miR-200b

Risk scores were then dichotomized about the median to create high and low risk groups for generating survival curves and Kaplan-Meier estimates. The miRNA risk score was used as a continuous variable in the multivariate analysis, with one risk unit increase equaling 9.5 miRNA risk score units (10.4% of the range).

Multivariable analysis was performed using age, pathologic T and N stage, histologic grade, post-operative CA 19-9, and surgical margin status in our institutional cohort.

### Validation datasets

We attempted to validate the association of miRNA risk score with overall survival using two independent datasets from the TCGA and Seoul National University (SNU). For the TCGA pancreatic cancer (PAAD) validation dataset [44], MiRseq expression data and clinical data for pancreatic adenocarcinoma primary solid tumor was downloaded from TCGA through http://firebrowse.org. MiRNA expression was provided as reads per million mapped, and 178 patients had usable data after filtering and normalization. As previously published, the SNU cohort had the miRNA gene expression profiling done by GeneChip^®^ miRNA 3.0 Array (Affymetrix, Santa Clara, CA) [28].

The same variables used in the original cohort for multivariable analysis were used for the TCGA dataset, with the exception of post-operative CA 19-9, which was unavailable. The miRNA risk score was again used as a continuous variable in the multivariate analysis, with one risk unit increase equaling 18.4 miRNA risk score units (5.4% of the range) for the TCGA cohort. Multivariable analysis was unable to be performed in the Korean dataset due to multiple unavailable clinical variables.

## SUPPLEMENTARY MATERIALS


