# Supplementary material for: A microRNA-based signature predicts local-regional failure and overall survival after pancreatic cancer resection
Source: Oncotarget. 2020 Mar 10;11(10):913–23. doi: 10.18632/oncotarget.27496 (PMC7075466; doi:10.18632/oncotarget.27496)
Supplement: Supplementary file 1 [file oncotarget-11-913-s001.pdf]

A microRNA-based signature predicts local-regional failure and overall survival after pancreatic cancer resection

SUPPLEMENTARY MATERIALS

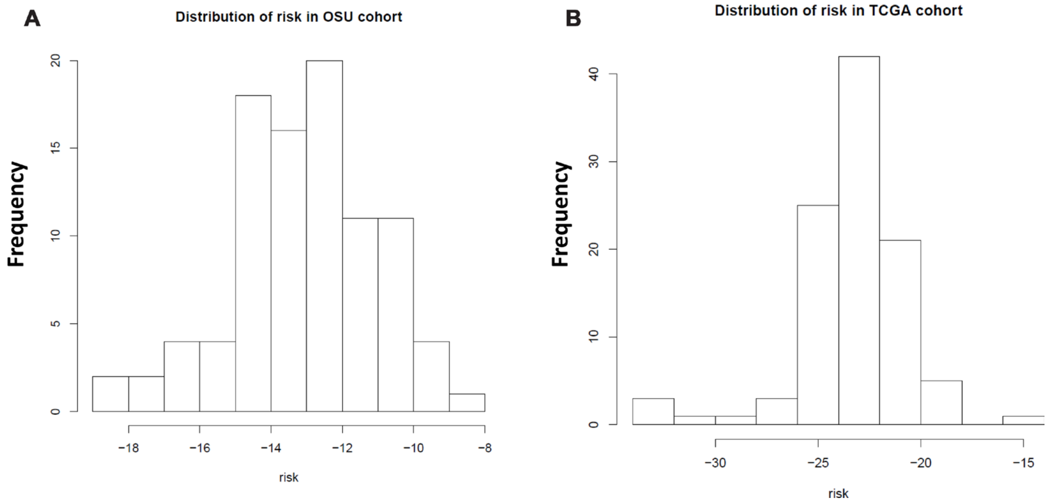

Supplementary Figure 1: Histogram of risk score per patient in the (**A**) OSU or (**B**) TCGA cohort.

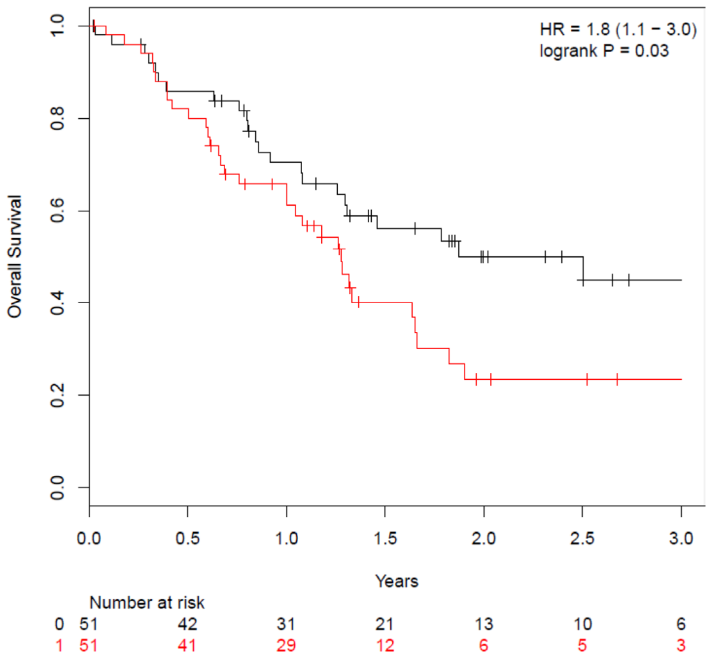

Supplementary Figure 2: Overall survival for high (red) versus low (black) risk groups in the TCGA cohort, only including patients who did not receive post-operative chemoradiation (*n* = 102 patients).

Supplementary Table 1: Multivariable analysis of TCGA patients for overall survival who did not receive post-operative chemoradiation (*n* = 102 patients)

Variable	Hazard ratio for OS	95% C.I.	*p*-value
miRNA risk score,continuous	1.15	1.03–1.30	**0.017**
Age, continuous	1.02	1.00–1.05	0.098
pTstage (T3-4 vs. T1-2 vs T1-2)	1.16	0.46–2.92	0.750
pNstage (1 vs 0)	1.30	0.61–2.77	0.498
Histologic grade (Grade3 vs Grade1-2)	1.71	0.97–3.01	0.064
Margin status (R1 vs R0)	2.30	1.28–4.13	**0.005**

Supplementary Table 2: Potential mechanisms and targets for miRNAs contributing to miRNA risk score

microRNA	Proposed target(s)	Proposed mechanisms
miR-29c	Specificity protein 1 (Sp1)	Suppresses EMT Inhibit migration/invasion
miR-125a	PI3K/AKT/mTOR	Suppresses proliferation/migration
miR-155	SOCS1and STAT3 MLH1	Inhibit proliferation/invasion
miR-200b	ERM ZEB1/ZEB2	Inhibit migration/invasion/proliferation SuppressesEMT

Supplementary Table 3: 30 miRNAs commonly linked to human pancreatic cancer based on literature search and used to generate the miRNA risk score which included miR-155, miR-29c, miR-125a, and miR-200b

Function	miRNA
Oncogenic (up-regulated)	10a, 15a, 21, 98, 99a, 100, 125b, 155, 221, 222, 223, 301a, 424, 497, 574
Tumor Suppressor (down-regulated)	Let-7b, 26a, 29c, 30a, 30d, 34a, 125a, 126, 141, 200a, 200b, 200c, 217, 375, 494

“Oncogenic” miRNAs are typically up-regulated in pancreatic cancer, while “tumor suppressor” miRNAs are typically down-regulated in pancreatic cancer.
